# How does one health fit into current academic frameworks? Insights from a SWOT analysis by the North America One Health University Network

**DOI:** 10.3389/fmed.2025.1686116

**Published:** 2025-12-09

**Authors:** Amanda M. Berrian, James Ferrara, Alice Matos, K. Paige Carmichael, Christopher W. Woods, Siddhartha Thakur, Tracey Goldstein

**Affiliations:** 1 Department of Veterinary Preventive Medicine, College of Veterinary Medicine, The Ohio State University, Columbus, OH, United States; 2 Department of Pathology, College of Veterinary Medicine, University of Georgia, Athens, GA, United States; 3 Departments of Medicine and Pathology, Duke University School of Medicine, Durham, NC, United States; 4 Department of Population Health and Pathobiology, College of Veterinary Medicine, North Carolina State University, Raleigh, NC, United States; 5 One Health Institute, Colorado State University, Fort Collins, CO, United States

**Keywords:** One Health, workforce development, education, core competencies, curriculum, training, SWOT analysis

## Abstract

**Introduction:**

One Health (OH) has gained support by health organizations, academics, and policymakers worldwide. To advance the approach across its broad use categories, workforce development has been a focus area, resulting in many versions of recommendations for OH core competencies and a multitude of training programs at various learner levels. Questions remain about strengths (and weaknesses) of available programs and employment prospects for graduates.

**Methods:**

At the inaugural meeting of the North America One Health University Network (NAOHUN) in 2024, over 90 university and partner agency representatives discussed these questions through a workshop-based SWOT analysis, describing strengths (S), weaknesses (W), opportunities (O), and threats (T) facing OH education programs. Participants were grouped according to the program type offered by their organization: professional, graduate, undergraduate, micro-credential, non-degree program, as well as no current offering. Participants recorded their inputs on a collaborative web-based platform which were then exported and analyzed using thematic analysis.

**Results:**

Discussions helped to elucidate internal and external factors that both help and hinder the design and delivery of OH education. Insights indicate that the number and diversity of OH educational programs have increased over time as has the interest from prospective trainees. Challenges remain, however, in program administration, maintaining collaborations, and marketing of skills to employers.

**Conclusion:**

These discussions will help to ensure sustainability and relevance of OH training programs. With the launch of NAOHUN, these insights will provide strategic direction for establishing universally accepted OH education competencies and other priority initiatives for network members.

## 1 Introduction

The concept of One Health (OH) – an integrated, unifying approach that aims to sustainably balance the health of people, animals, and ecosystems (1) – has evolved significantly over time. In the past decade, the number of scientific publications and scholars with a OH focus have surged (2, 3), as have the number and diversity of academic programs (4). Veterinary schools, where the One Health approach has long been incorporated into training and research, are now integrating more interdisciplinary elements and experiential learning opportunities into their curricula, reflecting a growing student interest in OH concepts (5). Similarly, medical and public health schools have increasingly embraced OH, particularly through interprofessional education (6).

Several important initiatives, as early as 2008, have attempted to define, synthesize, and update OH competencies and learning objectives (7, 8, 9); however, no accrediting body for OH degree programs currently exists since the prevailing consensus among the OH community has been that the concept is an inclusive interdisciplinary approach rather than a standardized discipline (4). Regardless, many OH training formats currently exist, from formal academic programs across student levels to informal and non-degree programs (10, 11). More recent developments in education include introduction of OH concepts into primary and secondary school curricula (12) as well as degree enhancements, such as certificates, which increase the accessibility and cross disciplinarity of OH training and skills development (13).

At a time when many countries are advancing their National One Health frameworks, supported by guidance from intergovernmental agencies (14), OH workforce development is a priority. In the U.S., the 2023 Consolidated Appropriations Act passed by Congress directed the CDC, in coordination with other federal agencies, to create its first ever National One Health Framework to Address Zoonotic Diseases and advance Public Health Preparedness in the United States (NOHF-Zoonoses) (2025–2029) (15). This framework presents goals and objectives for application of the OH approach, primarily through enhanced coordination, collaboration, and communication at the federal level but recognizing that its success requires robust partnerships with state, Tribe, local, and territorial (STLT), non-governmental organizations, academia, private sector, and relevant international partners. A key goal of the framework includes the support and expansion of efforts to develop a qualified OH workforce. Specific objectives include the identification of training opportunities to build critical OH competencies as well as the integration of OH into curriculums across all relevant disciplines.

Despite the traction of OH in workforce development priorities and the emerging coalescence around core competencies, OH academic programs and educational structures still appear divergent (16). Programs tend to be designed around strengths of the organizing institution, emphasizing subsets of OH rather than a comprehensive overview (17). Additionally, the various program types are often housed in traditional academic institutions that tend to operate in administrative and disciplinary siloes, which is counter to the interdisciplinary nature of OH (18). To determine the strengths and opportunities for improvement of OH educational programming in North America, the authors facilitated a discussion-based SWOT analysis (strengths, weaknesses, opportunities, threats) at the inaugural meeting of the North America One Health University Network (NAOHUN) in August 2024. As a newly formed organization, the mission of NAOHUN is to synergize activities of OH programs at North American universities to enhance collaboration, optimize communications, and amplify their impact. An enhanced understanding of current programs will provide strategic direction for OH education and training initiatives that can be prioritized by network members and working groups to continue the institutionalization of OH across various disciplines and sectors.

## 2 Methods

### 2.1 Participants

The SWOT analysis was conducted within the inaugural meeting of the North American One Health University Network (NAOHUN) on August 9, 2024 and hosted by Colorado State University (Fort Collins, CO, USA). For this initial planning meeting, invitees were established using a list of universities, state, federal and private organizations with OH programming maintained by the One Health Commission^1^ [a 501(c)(3) organization headquartered in North Carolina, USA], targeted internet searches, and personal referrals. This list, along with associated contacts, was used both to assess interest in establishing NAOHUN and to extend invitations to the planning meeting. Invitees were encouraged to share the information and invitation with colleagues who may not have been directly contacted.

Meeting attendees included over 90 in-person participants from 43 universities and partner agencies across the U.S. and Canada. Participants represented academic institutions (29 – U.S., 5 – Canada), as well as government organizations (4) (e.g., U.S. Centers for Disease Control and Prevention, U.S. Food and Drug Administration), private sector (2) (e.g., Merck Animal Health), and professional associations (3) (e.g., American Veterinary Medical Association; National Academy of Sciences, Engineering, and Medicine).

### 2.2 Data collection

To facilitate workshop preparation, a brief online (Qualtrics) survey was distributed to meeting registrants via email prior to the event, requesting information about the available program types at their institution or agency (e.g., One Health major/minor, certificate, seminar series) and student level(s) they serve (e.g., undergraduate, graduate, professional). This information was used to inform smaller discussion groups and the relative distribution of participants to each group.

During the workshop, participants could self-select which small group discussion they would like to contribute to, based on their familiarity and experience with the major OH education program types identified by the pre-workshop survey. Twelve small groups were formed, each with 6–10 participants to optimize discussion dynamics. The study team (AMB, KPC, CWW) moderated the discussion-based SWOT analysis to better understand current and future factors (both internal and external to the host organization) impacting OH education in North America. The SWOT tool was selected due to its ability to facilitate productive discussions and strategic planning, focused not only on current challenges but also future opportunities that can be explored in a collaborative environment. Participants were reminded that all experiences were valid and noteworthy to encourage robust dialogue. Small groups were provided 20 min of discussion time to explore strengths, weaknesses, opportunities, and threats associated with their chosen program type. Participants were invited to anonymously record their individual reflections and discussion summaries via Padlet, a collaborative web-based platform^2^. A separate Padlet was developed for each of the program types identified to assist with organization and interpretation of the statements. Following the small group discussions, a large group report out (10 min) served to summarize key themes.

### 2.3 Data analysis

Pre-workshop survey data were analyzed with descriptive statistics (frequency, proportion). For in-workshop data, qualitative analysis was performed by multiple members of the study team (AMB, JF, AM) to enhance reliability and internal validity. Raw data, downloaded from Padlet as an Excel file, were reviewed several times by each research team member. Once the team was familiar with the content, data analysis was conducted, facilitated by NVivo 14.23.0.

Data were analyzed using a combined deductive and inductive coding approach through the lenses of the Four Cs (communication, collaboration, coordination, and capacity building), which have been proposed as the mechanism for One Health operationalization (1).

For the purposes of this analysis, the following working definitions were used for each of the Four Cs, informed by current resources (14, 19–21):

- Communication – imparting or exchanging information (i.e., how information is shared, both internal and external to the organization)
- Collaboration – action of working together to produce or create something (i.e., how individuals work jointly toward a common goal)
- Coordination – sharing of effective governance (i.e., how a program is organized to enable effective collaboration)
- Capacity Building – process of developing or strengthening skills, abilities, processes, or resources (i.e., approaches by which individuals or organizations improve or increase their impact or capability)

During the analysis the study team allowed new codes to emerge from the data when information did not fit any existing codes or when more specific aspects were identified under the broader themes; in this case, subcodes were created. The data were then reanalyzed to verify that the new codes were attributed consistently throughout the process. The pre-existing response categories (strength/weakness/opportunity/threat and program type) were maintained in the analysis phase as these categories provided a degree of contextualization that was important to the interpretation. To visualize the distribution of responses by these pre-existing response categories and the Four Cs proposed by the authors, a matrix coding query was generated in NVivo. With this visualization, a gradient from red to green was used to indicate the lowest to highest percentages, respectively, within each column allowing for comparison across program types within each SWOT category. To summarize the data, a table was generated which listed all codes (main and child) generated, sample responses attributed to their program type, as well as total mentions by SWOT category.

## 3 Results

### 3.1 Pre-workshop survey

A total of 59 responses were received prior to the workshop, representing 46 unique universities/organizations in North America. Most (71%) indicated their university/organization had an active, formal OH educational program (10% indicated uncertainty). Graduate-level programs (e.g., MSc, MPH, PhD) were most common (*n* = 31), followed by professional (e.g., MD, DVM) and undergraduate programs (*n* = 24 and 15, respectively). Participants could select all applicable program types.

Based on these inputs, six small group discussion themes were developed to which the ∼90 meeting participants could contribute: undergraduate (U), graduate (G), professional (P) (veterinary, medical, etc.), micro-credentials/certificate (MC), non-degree programs (ND) (seminars/workshops), as well as no current offering (no program).

### 3.2 SWOT analysis

**FIGURE 1:**
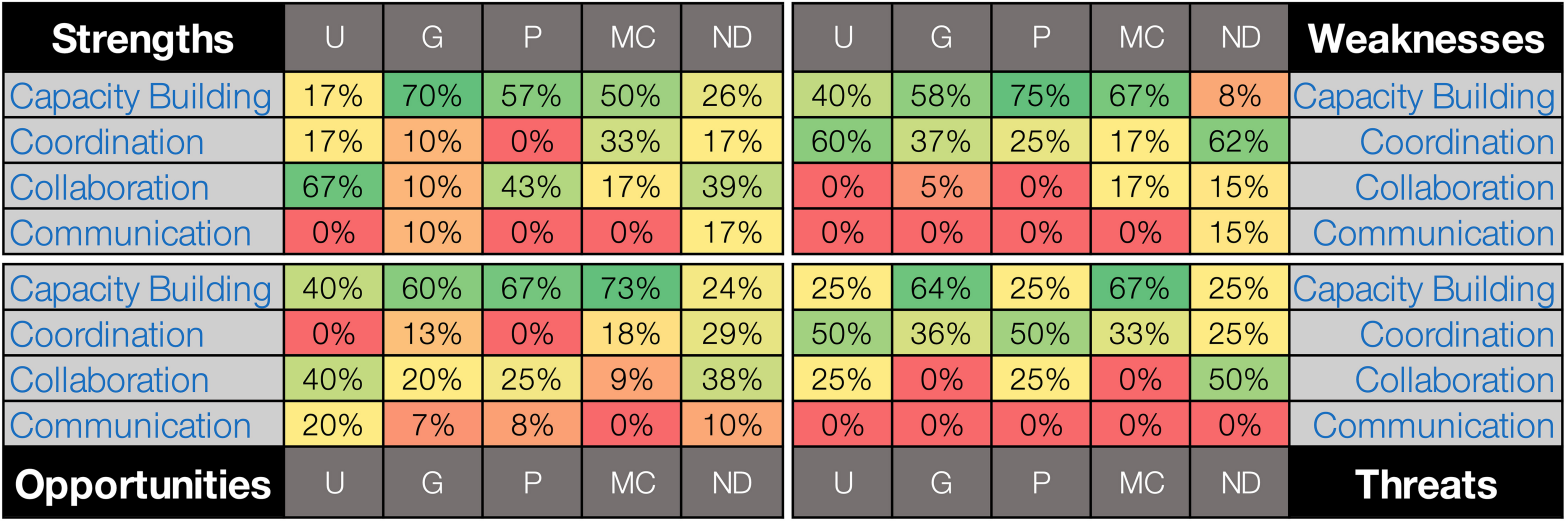
Coding matrix illustrating the distribution of NAOHUN workshop responses across the Four Cs of One Health implementation (capacity building, coordination, collaboration, communication), distributed by educational program type and SWOT category. Program types include undergraduate (U), graduate (G), professional (P), micro-credential/certificate (MC), non-degree program (ND). Each cell represents the percentage of responses coded under each “C” relative to the total responses for that program type and within a specific SWOT category. The matrix employs a red-to-green gradient, where green highlights the highest percentages within each SWOT category and red the lowest, facilitating comparison across program types within each SWOT domain.

Strengths, weaknesses, opportunities, and threats were identified across program types, with varying distributions across the Four Cs. For example, graduate program strengths strongly corresponded to capacity building, while undergraduate program strengths aligned mostly with collaboration. Coordination was identified as a prominent weakness and threat, primarily for undergraduate, professional, and non-degree programs (Figure 1). The coding matrix (Figure 1) shows these relationships visually with a red-to-green gradient, where green highlights the highest percentages within each SWOT category and red the lowest, facilitating comparison across program types within each SWOT domain.

A summary of the participants’ perceptions of their programs’ strengths, weakness, opportunities, and threats is provided below for each of the Four Cs. Representative quotes were chosen based on the relative number of responses in each SWOT domain and the distinct and clear nature of the idea presented (Supplementary Figure 1).

Capacity building (93 mentions) was the most frequently coded theme and included responses related to trainee professional development (i.e., desirable skills, positions) as well as the program itself (e.g., faculty expertise/availability, competency guidelines, program duration). Responses were primarily classified as opportunities (34 mentions) but were well represented across the SWOT categories and program types (Figure 1). Illustrative examples of participant responses, by program type, are:

**Table:** 

**Strengths (21 mentions)**
*“Students who really get it tend to be leaders within their broader programs because they learn multi-disciplinary communication”* (Graduate)
*“Seminar series turned into a course that brings multiple disciplines together and crowdsourcing of solutions, training grants”* (Non-Degree)
*“Great faculty interest”* (Graduate)
**Weaknesses (23 mentions)**
*“Seminars not leading to an “outcome” like a white paper or tangible product”* (Non-Degree)
*“Hard to train students both the breadth and depth in the field, especially for a 2-year degree”* (Graduate)
*“Many programs don’t have dedicated faculty to OH”* (Graduate)
*“Ability of faculty to develop courses”* (Microcredential)
*“Determining what methodological skills and not just “soft skills” are for One Health”* (Graduate)
**Opportunities (34 mentions)**
*“Integrating One Health principles into each course can be a promising approach”* (Professional)
*“Taking out biostats or regression skills and replace it with data management and dashboard building”* (Graduate)
*“Trainees bring a One Health vision and understanding back to their institution that tend to be more discipline-focused”* (Microcredential)
*“Leverage the breadth and vision of medical professional programs that recognize the scope of One Health and increase interprofessional programs to put these philosophies into practice”* (Professional)
*“Utilize faculty in different colleges across the university”* (Graduate)
**Threats (15 mentions)**
*“The trainees may not be able to change the siloes “ways of doing” in their institutions or jobs”* (Microcredential)
*“Does creating a separate major create another silo(?)”* (Undergraduate)
*“Integrating vertically can be challenging”* (Professional)

Coordination (48 mentions) included responses related to internal governance (i.e., how units/departments are organized to implement cross-disciplinary programs) and included administrative successes and challenges for OH training programs to include students from across colleges/schools and departments. In this theme, weaknesses/threats were mentioned more frequently than strengths/opportunities and perceived across program types, especially for undergraduate programs (Figure 1). Illustrative examples for each SWOT category include:

**Table:** 

**Strengths (8 mentions)**
*“List of elective courses that link into other departments, give some money back to those departments”* (Microcredential)
**Weaknesses (20 mentions)**
*“Not always a clear “home” department for One Health courses”* (Graduate)
*“Incorporate faculty teaching, research…responsibilities for promotion criteria when may span different departments or disciplines”* (Graduate)
**Opportunities (10 mentions)**
*“State funding to bring various departments of health together”* (Non-Degree)
*“Opportunity to restructure academia to recognize and support interdisciplinary work”* (Non-Degree)
**Threats (10 mentions)**
*“Sharing or sources of funds to start OH courses. Revenue sharing models may not allow resources needed to support courses”* (Undergraduate)

Collaboration (46 mentions) included responses related to student and faculty-level collaboration internal to the organization as well as with external partners. Participant discussions centered around strengths/opportunities rather than weaknesses/threats, which was particularly true for undergraduate programs (Figure 1). Illustrative examples for each SWOT category are:

**Table:** 

**Strengths (18 mentions)**
*“Opportunity for professionals from several disciplines to think about a problem with a One Health lens”* (Microcredential)
*“Field experiential training program allows flexibility to engage broadly with external partners and integrate many disciplines”* (Non-Degree)
*“Including faculty in One Health studies who do not realize their expertise IS a part of the One Health enterprise”* (Graduate)
*“Having students work not individually but together in multi-student teams from different disciplines on a OH project/problem/project…just as a professional would”* (Undergraduate)
**Weaknesses (4 mentions)**
*“Time to develop efficient teams (takes time to build trust to work together)”* (Non-Degree)
*“How do we involve…employers in the conversation”* (Microcredential)
*“Lack of university infrastructure for collaborative transdisciplinary teaching”* (Non-Degree)
**Opportunities (19 mentions)**
*“Inviting speakers and educators across disciplines”* (Undergraduate)
*“Could our professional organizations (e.g., AAVMC) support these initiatives as core?”* (Professional)
*“Identify a local issue – and bring students from across professions together”* (Professional)
**Threats (5 mentions)**
*“Perception of relevance of animals/veterinary medicine in the human medical world”* (Professional)
*“Not knowing what others are doing, lack of connectivity across universities to avoid reinventing the wheel”* (Non-Degree)

Communication (13 mentions) included responses related to how information is shared or exchanged both internal and external to the organization. Comparatively, the theme of communication was discussed less among the participants, which is apparent in the amount of red (low percentages) present in the communication theme for most program types and SWOT domains in Figure 1. No participant responses corresponding to “threats” were identified for this theme. Illustrative examples for the other SWOT categories are:

**Table:** 

**Strengths (5 mentions)**
*“Ability to advertise broadly across colleges”* (Non-Degree)
**Weaknesses (2 mentions)**
*“Language barriers across disciplines, cultures”* (Non-Degree)
**Opportunities (6 mentions)**
*“Opportunities to enhance communication across professions”* (Professional)

**FIGURE 2:**
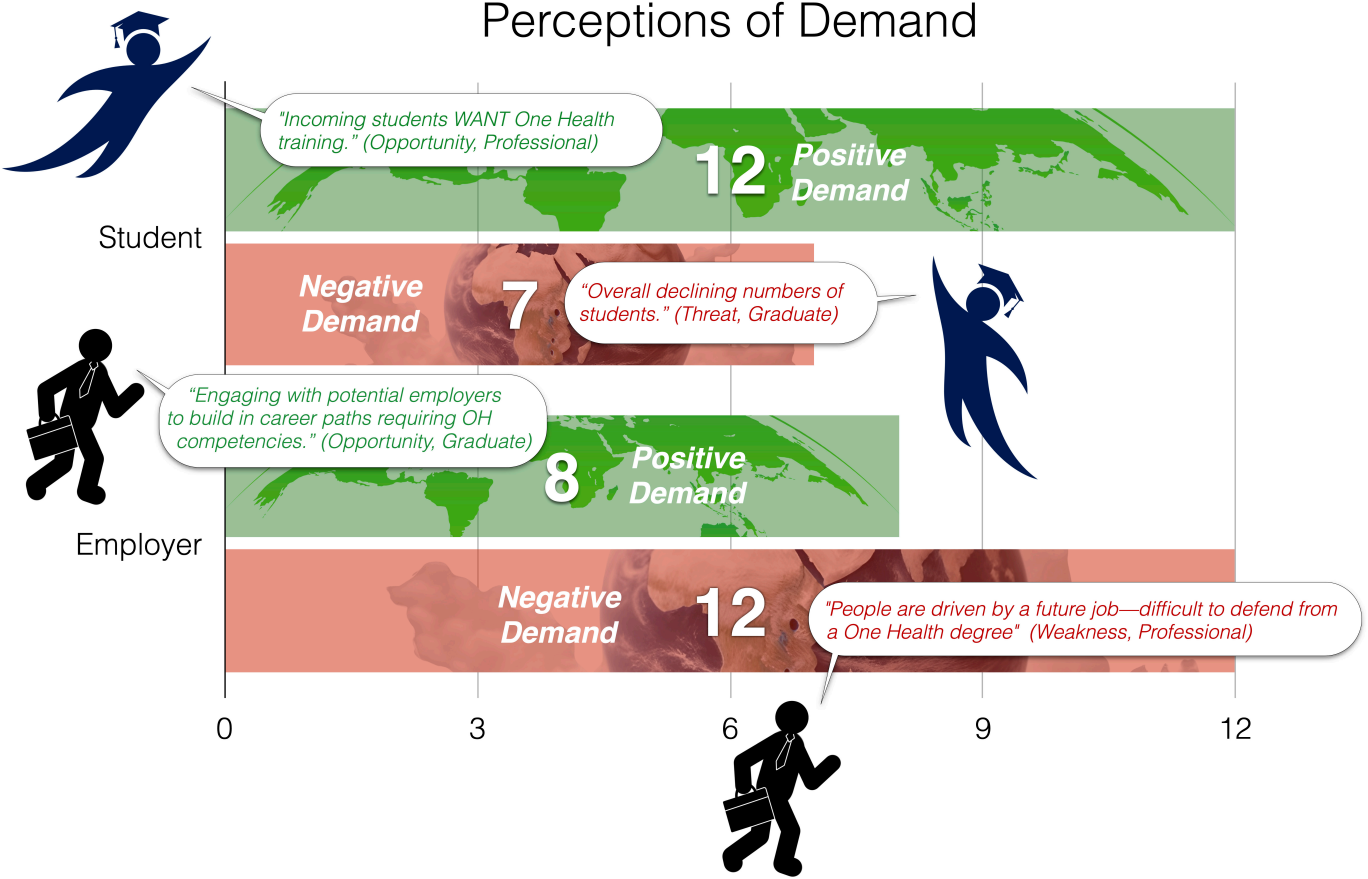
Perceptions of demand for One Health training programs from the student and employer perspective. The following visual assets are reprinted with permission from Adobe Stock (stock.adobe.com), licensed under Standard License: #1686413693, #1825793533, #926254801, #585051517.

Other main codes emerged that were not well encapsulated by one of the Four Cs. Most commonly (39 mentions), participants spoke about the demand for OH training programs by both employers (20 mentions) and students (19 mentions). Figure 2 depicts a visualization of the perception of demand from both the student and employer perspectives. Participants identified more strengths and opportunities (“positive perceptions”) related to student demand and more weaknesses and threats (“negative perceptions”) related to employer demand for One Health graduates.

An additional emergent code was related to flexibility (i.e., the ability for a training program to be modifiable or a student’s ability to adapt) (17 mentions). Most mentions were related to strengths (8) or opportunities (3); however, some participants reflected on the potential downsides of too much flexibility [*“Wide range of expertise based on electives students choose to take…What skills do all graduates need?”* (Graduate)] or not having enough free credit hours for students to customize their training with electives.

## 4 Discussion

It was evident from our SWOT analysis that OH educational offerings and program types target different needs and audiences, and they also have different strengths and weaknesses. From this perspective, there is a strong justification for academic institutions to offer multiple OH training program types (for example, a graduate and certificate program) that may target different audiences and/or primary disciplines. Organizations that offer non-degree OH training through seminars and workshops (such as federal agencies – e.g., CDC) can be valuable partners with academic institutions as they offer collaborative opportunities, including high-impact field experiences for students and professionals at different levels (22). Leveraging these partnerships to create communities of practice could offset some of the perceived weaknesses and threats of traditional training programs, such as lack of dedicated OH faculty and producing “marketable” graduates (23). Additionally, in the context of emergency preparedness and response, establishing relationships between academia and external organizations during “peacetime” can also translate to more effective collaboration when health emergencies or other crises occur (24). Networking, collaboration, and cross-sector and cross-disciplinary work are essential components of a successful OH training program and the programs themselves can be useful in building these networks, such as targeting working professionals through conferences, seminars, and other outreach initiatives (25).

One Health continues to be desirable among students at all levels; however, NAOHUN meeting participants perceived a mismatch between student and employer demand. This mismatch could be due to unclear career paths for OH graduates and emphasized by employers’ lack of understanding of the concrete, methods-based skills a OH education provides (26). Further, during the SWOT workshop debrief, a participant representing the employer perspective suggested that organizations may not explicitly use “One Health” in position descriptions although the desired skills and experience would be described in postings and well-aligned with those of a OH training program graduate.

From a program administration and coordination perspective, OH training programs can present challenges, particularly when operating under traditional university structures and hierarchies that lack incentives for cross-institutional collaboration and cross-posting of courses (23). Issues such as tuition revenue sharing, class and faculty scheduling, and administrative support are clear hurdles. Antiquated organizational structures can deepen and enforce silos by separating priorities and disincentivizing interdisciplinary work (18, 27). Students may experience difficulties in course registration and scheduling, and faculty with collaborative research and teaching may not be fully recognized by their home departments for career advancement and promotion purposes (23, 28). When a program is highly collaborative, cost and revenue sharing become more complex; however, the diverse nature of OH can translate to more innovative funding mechanisms that may be underexplored, such as foundations, private donors, or industry partnerships (29).

It was evident from the discussions that there is still debate about whether OH is a discipline and an approach or more squarely the latter. Furthermore, without a formal accrediting body, it is difficult to ensure consistent delivery of OH competencies across training programs – or if that is even desirable given the need to refrain from creating another siloed discipline (30). Many academic programs are already well-suited to include an OH approach; for example, professional programs (e.g., MD, DVM) teach an integrated problem-solving and reasoning approach, core elements of OH (31). However, one participant elucidated a potential threat, specific to professional training programs, which is the pressure from accrediting entities to train students to be “practice-ready” which can limit the scope of education and hamper one’s exploration of complementary fields and approaches. Interprofessional education, which is already taught in many human medical professions, is a promising framework to complement OH competencies and drive increased collaboration across medical and veterinary fields, in particular (23, 32). One participant offered the suggestion to increase interprofessional programs to put OH philosophies into practice.

Health-related fields are some of the most rapidly evolving, due to technology advancements, socioeconomic shifts, environmental factors, and other global changes. Preparing a future-ready health workforce will require continuous evaluation of core skills and adaptation of education and training programs to reduce the skills gap (33). Establishing a well-described competency framework for the North American context seems to be an important and well-positioned opportunity for NAOHUN, as is providing guidance on flexibly incorporating these competencies into new and existing programs and assessing their impact on the OH workforce. Based on the current strengths, weaknesses, opportunities, and threats related to OH education and training initiatives in North America, we provide the following recommendations for their design and implementation which address each of the Four Cs (Box 1):

Box 1 **Summary of recommendations for designing and implementing One Health training programs.**

- Ensure consensus among stakeholders and across various training programs on the core competencies required for One Health professionals; competencies should be reviewed and revised regularly to reflect current and emerging trends and regional priorities *(Capacity Building, Collaboration)*
- Assess employer needs to align One Health education with job market skills and demands; increase engagement with employers to ensure alignment and employability of graduates (“training-to-career pipeline”) *(Capacity Building, Collaboration)*
- Integrate authentic student assessments into learning activities to have students produce tangible and realistic outputs that utilize technical and functional competencies, such as policy briefs, tools, and guidelines *(Capacity Building)*
- Bring together internal and external stakeholders (private and public sector) to identify innovative solutions for coordination challenges, such as lack of funding and faculty/instructor shortages *(Collaboration, Coordination)*
- Evaluate administrative systems and structures to ensure cross-listing of courses and collaborative, inter-disciplinary research and teaching are incentivized *(Coordination, Communication, Collaboration)*
- Provide students with experiential learning opportunities, fieldwork and capstone projects to increase engagement with partner organizations and demonstrate real-world application *(Capacity Building, Collaboration)*
- Consider co-production and sharing of educational resources (e.g., case studies) across programs and institutions to enhance collaboration, relevance, and uptake *(Collaboration, Capacity Building)*

While previous research on OH education has identified gaps and recommendations for core competencies (4), this SWOT analysis uncovered additional themes related to employer demand and career outlook, marketing, and institutional policies and administration that can guide the evolution of OH educational programming to ensure their sustainability and relevancy in the future. Ensuring the alignment, relevance, and sustainability of OH education programs will be increasingly important, especially as the number of available programs continue to grow. By conducting the analysis in this setting with broad stakeholder participation, we can more effectively leverage strengths and mitigate risks across the continuum of OH education delivery, helping to ensure the sustainability and relevancy of these programs in the future.

## Data Availability

The original contributions presented in this study are included in this article/Supplementary material, further inquiries can be directed to the corresponding author.

## Footnotes

1
https://www.onehealthcommission.org/en/resources__services/whos_who_in_one_health/

2
https://padlet.com/

## Supplementary material

Supplementary Figure 1 Themes coded during qualitative analysis with descriptions of each main code and child code(s) as well as counts of each code and sample responses. Strengths, green; weaknesses, red; opportunities, blue; threats, purple. U, undergraduate; G, graduate; P, professional; MC, micro-credential/certificate; ND, non-degree/workshop/seminar. Counts are also separated into total count for the code, “addends” or counts of the child codes.
