# Supplementary figures and images for: How does one health fit into current academic frameworks? Insights from a SWOT analysis by the North America One Health University Network
Source: Front Med (Lausanne). 2025 Dec 9;12:1686116. doi: 10.3389/fmed.2025.1686116 (PMC12723863; doi:10.3389/fmed.2025.1686116)

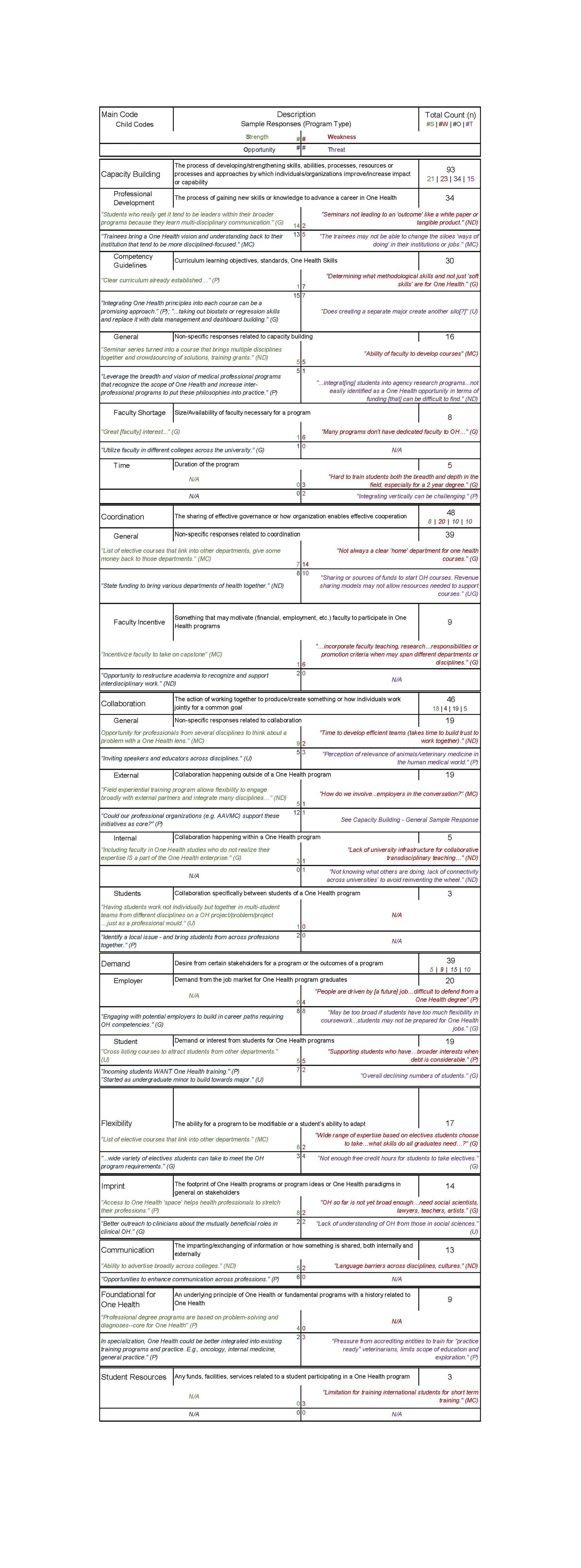

Supplement: Supplementary Figure 1 — Themes coded during qualitative analysis with descriptions of each main code and child code(s) as well as counts of each code and sample responses. Strengths, green; weaknesses, red; opportunities, blue; threats, purple. U, undergraduate; G, graduate; P, professional; MC, micro-credential/certificate; ND, non-degree/workshop/seminar. Counts are also separated into total count for the code, “addends” or counts of the child codes. [file Image_1.jpeg]
